# From Entry to Outbreak in a High School Setting: Clinical and Wastewater Surveillance of a Rare SARS-CoV-2 Variant

**DOI:** 10.3390/v17040477

**Published:** 2025-03-27

**Authors:** Sven Sachse, Ivana Kraiselburd, Olympia Evdoxia Anastasiou, Carina Elsner, Sarah Christina Goretzki, Stefan Goer, Michael Koldehoff, Alexander Thomas, Jens Schoth, Sebastian Voigt, Rudolf Stefan Ross, Ulf Dittmer, Folker Meyer, Ricarda Maria Schmithausen

**Affiliations:** 1 Institute for Artificial Intelligence (IKIM), University Hospital Essen, University of Duisburg-Essen, 45131 Essen, Germany; sven.sachse@uni-due.de (S.S.); ivana.kraiselburd@uk-essen.de (I.K.); 2 Department of Hygiene and Environmental Medicine, University Hospital Essen, University of Duisburg-Essen, 45147 Essen, Germany; 3 Center for Water and Environmental Research, University of Duisburg-Essen, 45141 Essen, Germany; 4 Institute of Virology, University Hospital Essen, University of Duisburg-Essen, 45147 Essen, Germany; 5 Department of Pediatrics I, Neonatology, Pediatric Intensive Care, Pediatric Infectiology, Pediatric Neurology, University Hospital Essen, University Duisburg-Essen, 45147 Essen, Germany; 6 Emschergenossenschaft Lippeverband (EGLV), 45128 Essen, Germany

**Keywords:** SARS-CoV-2, outbreak investigation, wastewater

## Abstract

In December 2021, an outbreak of the SARS-CoV-2 B.1.640.2 variant, potentially originating from Cameroon, was investigated among schoolchildren in Germany. The index case, an adult who had recently returned from a three-week stay in the Republic of Congo, introduced the variant into a school setting via their children, resulting in subsequent transmission within the school and ultimately to a hospital ward. Whole-genome sequencing of viral samples identified both B.1.640.1 and B.1.640.2 lineages. This outbreak highlights the unpredictable nature of emerging SARS-CoV-2 variants and emphasizes the importance of early detection and containment to mitigate transmission to high-risk populations. Notably, wastewater surveillance detected the variant during the study peri-od, reinforcing the utility of wastewater-based epidemiology as a complementary tool for the early warning and containment of novel variants. These findings underline the critical need for timely research and adherence to quarantine measures to enhance outbreak control efforts.

## 1. Introduction

The COVID-19 pandemic highlighted the importance of understanding viral variants and their potential introduction into new geographical areas and vulnerable institutions. Due to their impact on transmission rates, immune escape, etc., the emergence of new viral variants poses a significant challenge in containing and managing the COVID-19 pandemic. This study investigates a COVID-19 outbreak in a high school in Germany caused by the B.1.640.2 variant, a novel variant at a time when the Delta (B.1.617.2) variant represented 98% of infections in Germany. This study aims to shed light on the introduction and transmission dynamics of the B.1.640.2 variant in this specific setting and its impact on various institutions. The findings of this study contribute to our understanding of the unpredictability of SARS-CoV-2 variants and the importance of early detection and containment measures to prevent further spread and mitigate the impact on vulnerable populations. Monitoring emerging viral variants such as B.1.640.2 is an effective tool for responding to and managing pandemics. Accurate surveillance and research on viral variants like B.1.640.2 are crucial for public health authorities to make informed decisions regarding testing, diagnostics, treatment, and vaccine development. It is also crucial to implement public health policies, strategies and interventions to prevent the spread of COVID-19, especially in vulnerable institutions such as schools and hospitals. In addition, it plays a pivotal role in understanding the transmission dynamics and potential impact of these variants, as well as informing targeted interventions and strategies to mitigate their spread and protect vulnerable populations. Furthermore, our study demonstrates that complementing clinical testing with wastewater-based epidemiology (WBE) will enhance our ability to observe emerging variants, building the basis for earlier and more targeted mitigation measures.

### Background

SARS-CoV-2 variants have repeatedly emerged over the last years and have since become a major health concern. Thus, the WHO monitors all emerging SARS-CoV-2 virus variants in the world. While some assume that viruses mutate and become more infectious and less deadly over time [1,2,3], current research demonstrates that the human immune status underlies this observation [4]. Novel variants might gain the ability to evade the host’s immune defenses and therefore need to be under surveillance, as variants with a high number of mutations are especially alarming due to their unexpected behavior, making them much more transmissible and capable of spreading more rapidly. Omicron followed this pattern. The original Omicron variant (BA.1/B.1.1.529) was first reported by the WHO and uploaded to the GISAID disease variant database on 23 November 2024, with more than 300,000 sequences in the database to date [5,6,7].

However, another variant of interest, Pangolin lineage B.1.640, predates Omicron. The world was alarmed because both variants had many mutations in common, making them much more rapidly transmissible [8,9]. The question was whether and to what extent these two variants would spread and shape the spread of infection. This second variant of interest was first identified in France in April 2021, and later in the Republic of Congo (Brazzaville) in September, and was further involved in a cluster of cases in southeastern France from a vaccinated traveler returning from Cameroon around mid-October 2021 [9]. Until November 2021, a total of 12 cases were detected in the region, and the variant was named B.1.640 and uploaded to GISAID on 4 November 2021 [10].

In addition, French researchers detected an atypical combination of mutations within the B.1.640 variant at the beginning of December 2021 in samples from twelve people infected with Corona, including five children under the age of 15 [10]. The atypical variant was identified by screening with qPCR assays from the Delta (L452R-positive) and Omicron (L452R-negative and negative for S gene detection with the TaqPath COVID-19 assay) variants that co-circulated in Europe at that time [11].

This new lineage, known since 12 December 2021, was named B.1.640.2. It is a phylogenetic sister group to the B.1.640 lineage, renamed to B.1.640.1. B.1.640.2 has up to 46 mutations that are already known from other virus variants. These include mutation N501Y, which is known from the Alpha, Beta, Gamma, and (other) Omicron variants. It enables the coronavirus to bind more strongly to its molecular target on human cells, making it more easily transmissible [12]. The mutations suggest that B.1.640.2 may be more contagious than the original virus discovered in Wuhan. However, at that time, it remained unclear whether this virus variant would prevail. By that time, B.1.640.2 had been detected in only a few sequencing runs [10].

At that time Omicron was about to take over as the predominant variant circulating worldwide but had not yet started to dominate as it would a month later, being responsible for over 75% of sequences reported to GISAID [13]. Even if only a few cases of B.1.640.2 were present, this case report outlines how the virus variant could spread to a high school in Western Germany causing a mid-scale outbreak cluster.

## 2. Materials and Methods

### 2.1. Setting

On 6 December 2021, the routine screening of healthcare workers at a maximum care hospital in Western Germany led to the detection of the B.1.640.2 sequence. The variant was first identified in France in April 2021 and traced to Congo. According to the Robert Koch Institute, this was only the second identification of this variant in Germany.

The case involved an unvaccinated female nurse working in the neonatal ICU who was exempt from vaccination for medical reasons, necessitating strict adherence to hygiene protocols and regular PCR testing. She developed mild respiratory symptoms on December 5, one day before her positive test.

The main goal was to prevent the spread of this variant at the hospital and to trace the transmission chain across the hospital and a high school in the Ruhr metropolitan region, where additional cases had been identified. The study period spanned from the initial detection on 6 December 2021, until outbreak containment in mid-January 2022.

This period included recruitment, data collection and follow-up.

### 2.2. Data and Investigation Procedures

Recruitment was performed by passing on information to the school, teachers, and parent groups. On this basis, parents and positive individuals contacted us independently and voluntarily. A comprehensive anamnesis was conducted by a hygienist, complemented by targeted virological testing.

The inclusion criteria were positivity and close contact with affected class members and/or healthcare workers. Close contacts were defined as persons with more than 15 min of face-to-face contact indoors and outdoors and family/household members with a confirmed COVID-19 case during the infectious period.

According to the principles of the Declaration of Helsinki and the approval of the local institutional research ethics board (ethics vote 468/20), informed consent was obtained from all participants, and in the case of minors, through the signature of at least one parent.

Participation criteria were recorded in personal interviews following a structured questionnaire. All participants who agreed to participate were visited at their homes. Data on sociodemographic factors, exposures, medical history, comorbidities, vaccination schedules, and epidemiological data were collected for all participants. Furthermore, study participants were asked to provide nasopharyngeal and deep throat swabs.

The participants were sampled up to 3 times within a fortnight at various time points during periods of illness. Initially, all children from the affected fifth grade who became ill and/or tested positive with a rapid test were tested by the volunteer fire department on 10 December 2021. In this step, nasopharyngeal and deep throat swabs were collected using M 40-compliant Transwab systems (Medical Wire, Corsham, UK).

In the next testing steps—(i) one week later, December 17, (ii) on December 23, (iii) on December 27, and/or (iv) in mid-January 2022—throat swabs for virus diagnostics were obtained using liquid medium swabs (UTM, Befl. swabs, Minitip m. 3 mL UTM (16 × 100 mm) COPAN, Brescia, Italy). The swabs were stored in UTM Viral Stabilization Media at 4 °C at the study center for up to four hours. Swabs were resuspended at a maximum of 6 h after collection, and all laboratory analyses were performed within 48 h.

### 2.3. Laboratory Analyses

#### 2.3.1. Detection of SARS-CoV-2 RNA

SARS-CoV-2 RNA was qualitatively detected using a real-time PCR SARS-CoV-2 AMP Kit (Abbott Diagnostics, Wiesbaden, Germany), a dual-target assay for the viral RdRp and N genes, which was run on the Alinity m system with automated sample preparation (Abbott Molecular, 2021). Alternatively, viral nucleic acids were extracted using an MagNA Pure 96 DNA and Viral NA Small Volume Kit (Roche Diagnostics, Mannheim, Germany), and then reverse-transcribed and amplified using a RealStar SARS-CoV-2 RT-PCR Kit 1.0 (Altona Diagnostics, Hamburg, Germany) on the CFX 96 system (Bio-Rad, Feldkirchen, Germany).

#### 2.3.2. Molecular Characterization of SARS-CoV-2 Isolates

Melting curve assays: Probe-based melting curve analyses were used to screen for common mutations in the viral genome and to provisionally characterize SARS-CoV-2 isolates. The following VirSNiP SARS-CoV-2 Spike assays, provided by TIB MOLBIOL (Berlin, Germany) and run on a Roche LC 480 System, were used: del HV69/70, S371L/S373P, E484K, and N501Y. In the resulting curves, a higher melting peak generally indicates the presence of the mutated variant, whereas wild-type sequences show a peak at lower melting temperatures.

Sanger sequencing and data analysis: Extracted viral RNA was amplified using the Super-Script™ III One-step-RT-PCR System with Platinum™ Taq DNA Polymerase (Invitrogen, Waltham, MA, USA) with specific primers from two regions of the SARS-CoV-2 spike gene (Supplementary Figure S1) and the following cycling conditions: initial RT step 30 min at 55 °C followed by an initial denaturation for 2 min at 94 °C, 35 cycles of denaturation 15 s at 94 °C, annealing 30 s at 60 °C, and elongation 1 min at 68 °C, with a final elongation of 5 min at 68 °C. In a second step, nested PCR with gene specific primers (Supplementary Figure S1) was performed using the Platinum Taq DNA Polymerase HF (Invitrogen) with the following cycling conditions: initial denaturation of 2 min at 94 °C followed by 35 cycles of denaturation for 15 s at 94 °C, annealing for 30 s at 60 °C, and elongation for 1 min at 68 °C, with a final elongation of 5 min at 68 °C. The PCR amplicons were quality-checked on a 1% agarose gel, purified using the QIAquick PCR purification Kit (Qiagen, Hilden, Germany) and sent for Sanger sequencing to Eurofins Genomics (Ebersberg, Germany).

The sequence editing, generation, and translation of the consensus sequence into the corresponding amino acid sequence were performed using Geneious Pro 5.1.7. A multiple-sequence alignment was performed by Clustal Omega version 2.1 using the SARS-CoV-2 isolate Wuhan-HU-1 (NC_045512.2) as a reference. The visualization of the Clustal Omega alignment was carried out with a Highlighter analysis using the Los Alamos National Laboratory pathogen database. Initial manual lineage prediction was performed based on mutations identified in a multiple-sequence alignment using out-break.info [14]. Whole-genome sequencing (see below) later confirmed the initial lineage assignment.

A phylogenetic tree was inferred using the Maximum Likelihood method and the Tamura-Nei nucleotide substitution model based on the receptor binding domain of the viral spike gene (nucleotides 963 to 1737). The tree was drawn to scale, with branch lengths measured in the number of substitutions per site. Statistical robustness was tested using the bootstrap approach with 1000 replicates. Analysis was conducted in MEGA, version 11 [15].

The partial SARS-CoV-2 spike gene sequences obtained from the infected individuals in this study were deposited into the GISAID database under accession numbers EPI_ISL_10033340–EPI_ISL_10033367.

#### 2.3.3. Next-Generation Sequencing and Data Analysis

SARS-CoV-2 whole-genome sequencing was performed using a tiled amplification protocol [14]. For library preparation, the EasySeq™ SARS-CoV-2 Whole Genome NGS Sequencing kit (Nimagen) was employed after generating cDNA from the extracted viral RNA using the LunaScript RT SuperMix Kit (New England Biolabs (NEB, Ipswich, MA, USA). Normalized and pooled libraries were sequenced on an Illumina MiSeq instrument employing V2 chemistry (300 cycles).

Reads with an average Phred quality below 20 and a length below 30 base pairs were excluded, enabling downstream analysis. Subsequently, data analysis was performed with the UnCoVar bioinformatic pipeline for reconstructing whole viral genomes [16]. UnCoVar performed a series of QC steps, initially attempted de novo assembly, and then resorted to co-assembly for recalcitrant samples; it subsequently used pangolin [17] and Kallisto [18] matching to GISAID [19] to obtain lineage calls. Additionally, Freebayes [20], Delly [21], and Varlociraptor [22] were utilized for variant calling.

The full-length SARS-CoV-2 sequences obtained from the infected individuals in this study were deposited in the GISAID database under accession numbers EPI_ISL_9402306–EPI_ISL_9402318.

#### 2.3.4. Viral Detection in Wastewater

During this study, wastewater samples were collected within the metropolitan area Ruhr and processed for SARS-CoV-2 detection, as described [23]. The sampling area comprises both the high school and maximum care hospital described in this work.

Untreated wastewater samples spanning 24 h periods were filtered through 0.45 µm electronegative filters for viral concentration followed by automated RNA extraction with the innuPREP AniPath DNA/RNA Kit on an InnuPure C16 system (Analytik Jena, Jena, Germany).

Library preparation and viral genome sequencing were conducted as outlined in the previous section.

Viral variants were identified with a modified version of UnCoVar [16], based on the detection of variant-specific mutations without the attempt at genome assembly.

## 3. Results

### 3.1. Laboratory Results

Six full-length sequences were obtained for six samples, which were classified using pangolin (v3.1.20) as B.1.640.1. However, a deletion of nine amino acids in the S protein at locations 135–144 was observed, which [9] describes as indicative of lineage B.1.640.2. Kallisto also supported this assignment, as the majority of reads were identified as B.1.640.2 in six samples.

According to the outcome of the melting curve analyses, the SARS-CoV-2 isolates harbored by the infected schoolchildren and their relatives contained neither spike gene mutations S371L/S373P (Figure S1A) nor amino acid exchange L452R (Figure S1B). Therefore, they most likely did not represent the emerging Omicron or the dominant Delta variants that were the focus of attention in Germany in December 2021.

Furthermore, the isolates lacked deletion HV69/70 and mutation E484K but contained the N501Y mutation (Figure S1C), so they could not be clearly assigned to the SARS-CoV-2 Alpha, Beta, or Gamma lineages. Consequently, the results of the melting curve assays remained inconclusive.

**Figure 1:**
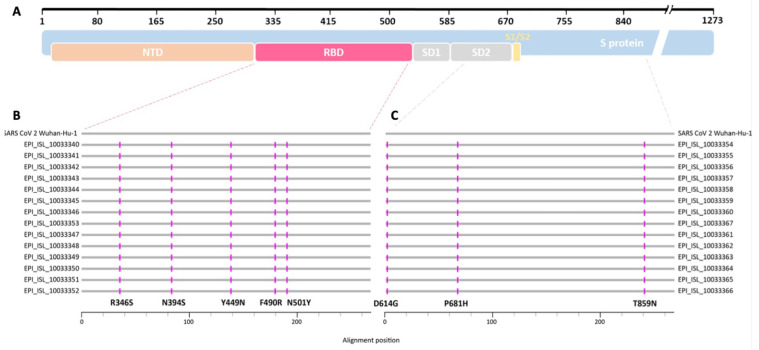
(**A**) Schematic overview of the SARS-CoV-2 spike protein (blue) and locations of selected protein domains such as N-terminal domain (NTD, orange), receptor binding domain (RBD, red), subdomains 1 and 2 (SD1, SD2, gray), and the S1/S2 cleavage site (yellow) (adapted from https://covdb.stanford.edu/. Last accessed: 17 February 2022). (**B**,**C**) Protein alignment of the sequences resulting from the SARS-CoV-2 B.1.640.1 outbreak in a secondary school. Horizontal gray lines correspond to the consensus sequence of each patient (GISAID database accession numbers given) and the SARS-CoV-2 Wuhan-Hu-1 (NC_045512.2) reference sequence, respectively. Amino acid exchanges are depicted in pink. (**B**) Alignment of about 268 amino acids covering the RBD of the SARS-CoV-2 spike protein (red dashed line). (**C**) Alignment of about 275 amino acids covering the SD2 and part of the C-terminus of the protein (gray dashed line).

The direct sequencing of two 804 bp and 825 bp long nucleotide fragments from the SARS-CoV-2 spike gene and subsequent translation into the corresponding amino acid sequences revealed the presence of eight mutations when compared to the wild-type Wuhan-Hu-1 strain (Figure 1).

**Figure 2:**
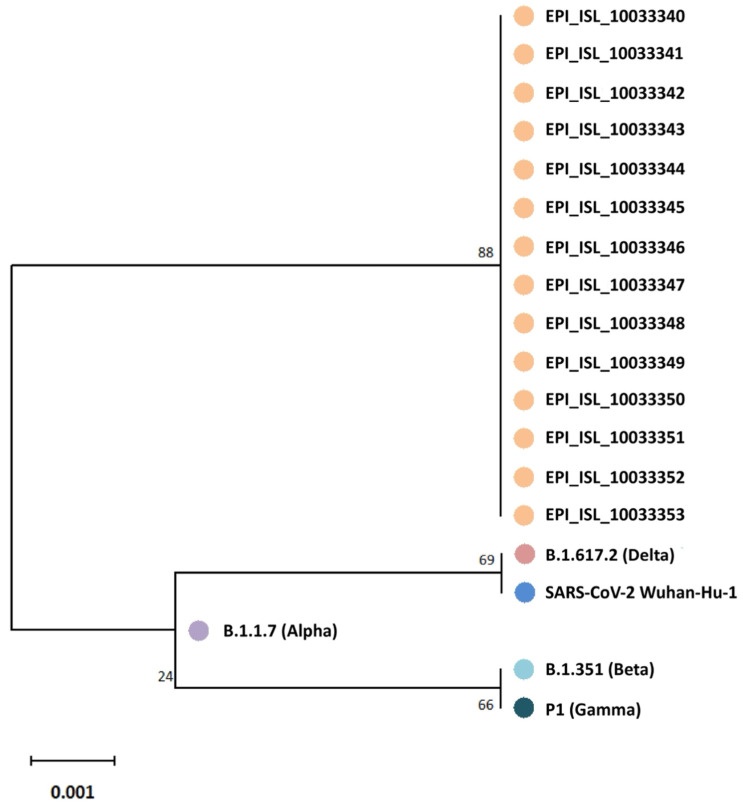
Phylogenetic tree based on partial SARS-CoV-2 spike gene RBD sequences (nucleotides 963 to 1737) from 14 infected schoolchildren and their relatives (GISAID database accession numbers given). Five epidemiologically unlinked isolates were included as references: EPI_ISL_3115625 (Alpha), EPI_ISL_1282471 (Beta), EPI_ISL_1849483 (Gamma), EPI_ISL_8038881 (Delta), and NC_045512.2 (SARS-CoV-2 Wuhan-HU-1). The tree is drawn to scale, with branch lengths representing the number of substitutions per site. Codon positions included are first, with 258 amino acid positions in the final dataset.

This characteristic pattern of amino acid exchanges had already been reported for variants from the former SARS-CoV-2 B.1.640 lineage, which was renamed to B.1.640.1 [9]. Analyses of full-lengths sequences obtained by next-generation sequencing confirmed and extended the findings from [9]. Additionally, the formation of a so-called monophyletic group in a tree based on partial SARS-CoV-2 spike gene RBD sequences, which reconstructs the evolutionary history of the viral isolates (Figure 2), shows that the children and their relatives were infected in a single-source outbreak by a yet rare SARS-CoV-2 B.1.640.1 variant, which had possibly originated in the Republic of Congo [9].

### 3.2. Detailed Outbreak Course

The outbreak described here was first identified on 6 December 2021, following a positive SARS-CoV-2 B.1.640.2 test result in a nurse. The investigation suggested that she likely contracted the infection from her teenage daughter, who had shown symptoms three days earlier and tested positive at her school. Due to the nurse’s absence from the neonatal ward during this period, secondary hospital transmission was ruled out.

Following an interview with the initial case, we focused on tracing contacts within the school setting. Between 6 December and 31 December 2021, a total of 20 individuals, including classmates and household contacts, participated in the study, all providing informed consent. Data collection included face-to-face interviews conducted by a hygienist, covering symptom history, clinical signs, prior laboratory results, and potential COVID-19 exposure within familial and social networks. We also collected student schedules to map potential in-school transmission pathways.

The first follow-up testing, conducted on December 13, confirmed the isolation of all SARS-CoV-2-positive students and their contacts. Based on interviews with the affected students and their families, we hypothesized that the introduction of the B.1.640.2 variant stemmed from a classmate who was in close contact with the outbreak’s index case. This index case, an adult diagnosed with SARS-CoV-2 via qPCR on November 26, had recently returned from a three-week stay in the Republic of Congo, where he was likely infected with the IHU variant. His initial symptoms appeared on November 25, coinciding with his return journey.

**Figure 3:**
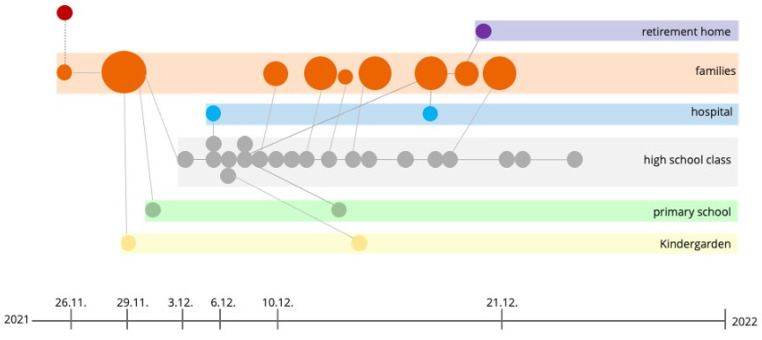
This picture provides a timeline and transmission pathway of the SARS-CoV-2 B.1.640.2 variant outbreak across multiple settings, including family units, a hospital, a high school, a primary school, kindergarten, and a retirement home. The outbreak originated on 26 November 2021 and was traced back to an individual returning from the Republic of Congo. Circles represent confirmed cases, with larger circles indicating clusters of infections. Transmission links between family members and within institutional settings are shown, demonstrating how the virus spreads through close contacts in schools, a healthcare setting, and a family network. Preventive measures and Christmas holidays achieved containment.

Data from pharyngeal swabs and questionnaires were categorized into three groups for analysis: (i) Full Sets (sample, survey, follow-up interview), (ii) Half Sets (sample and survey), and (iii) Sample or Questionnaire Only. Detailed analyses were performed using only the Full-Set data. The outbreak course is summarized in a flow diagram illustrating transmission within different institutions (Figure 3).

### 3.3. Symptoms and Vaccination Status

Clinical data were available for all confirmed COVID-19 cases linked to the school outbreak and affected healthcare workers. Eight tested positive via PCR at the school between 1 December and 13 December 2021. An additional 12 cases, initially negative, turned positive between 13 December and 28 December due to close contact with infected students. Altogether, 20 cases were confirmed, none of which required hospitalization or resulted in fatality.

Mild or asymptomatic courses were observed in 59% of cases. Among symptomatic individuals, the most common symptoms included fatigue (73%), rhinorrhea (68%), sore throat (38%), headache and cough (35%), fever, gastrointestinal symptoms (21%), and loss of taste or smell (12%). No complications, hospitalizations, or severe outcomes were recorded.

Regarding vaccination status, five students aged 12 had received one or two doses of the BNT162b2 vaccine or had previous SARS-CoV-2 infections, while others had no prior COVID-19 history.

### 3.4. Epidemiological Links

#### 3.4.1. The Index Case and Entrance to Germany

The index case was likely infected with the B.1.640.2 variant during a professional stay in the Republic of Congo. Upon returning to Germany on 26 November 2021, the individual developed mild respiratory symptoms en route, which led to a SARS-CoV-2 diagnosis via real-time qPCR testing on 26 November. Given the timeline and the case’s travel history, alternative routes of B.1.640.2 introduction into Germany were carefully examined and excluded based on epidemiological and travel data. No other exposures were identified in Germany before symptom onset, reinforcing that the variant was likely contracted during his stay abroad.

This scenario emphasizes the potential for travel-related transmission and the need for the vigilant surveillance of travelers returning from regions with emerging SARS-CoV-2 variants. The introduction of B.1.640.2 highlights how even rare variants can establish footholds in new regions. It underscores the importance of early detection and rapid containment strategies to prevent further spread within high-contact settings like schools and healthcare facilities.

#### 3.4.2. Exclusion of Cases and Transmission Events in a School

Initial transmission within the school cluster appeared to occur during break periods, with the earliest identified transmission event traced to a school break on 29 November. According to student reports, nasal swab testing was conducted following this break, revealing subsequent cases. Potential transmission settings, such as staff meetings or parent-teacher events, were excluded from consideration, as no COVID-19 cases were linked to these gatherings.

Several cases were ruled out based on symptom onset and sequencing data. One infected student, an affected sibling and their household contacts without sequencing data were classified as part of a separate outbreak due to distinct symptom timelines. Another sibling, attending kindergarten in a separate outbreak, was similarly excluded. Two students with early symptom onset were also ruled out as index cases. Sequence analysis confirmed that one of these students carried a different lineage (B.1.258), disqualifying them from association with the B.1.640.2 outbreak.

To further confirm the outbreak lineage, a phylogenetic tree was constructed using a custom pipeline and 9471 selected SARS-CoV-2 sequences from both German and international databases (see Methods Section 2). This analysis helped delineate the specific lineage relationships and confirmed the outbreak’s association with the B.1.640.2 variant, supporting targeted containment efforts within the school setting.

#### 3.4.3. Spillover from School to Hospital

Regarding infections in the hospital from an asymptomatic healthcare worker, while on weekend duty, this nurse performed blood draws across two hospital wards where mask wearing was not mandatory. The following Monday, the nurse developed symptoms, including fever and cough, and tested positive for COVID-19. Within 24 h of diagnosis, the employee entered home isolation. However, 27 patients across the affected wards developed confirmed COVID-19 infections over the next ten days. After these initial cases, no further in-house transmissions were observed following the rigorous application of a standardized hygiene protocol, highlighting the effectiveness of rapid containment measures.

**Figure 4:**
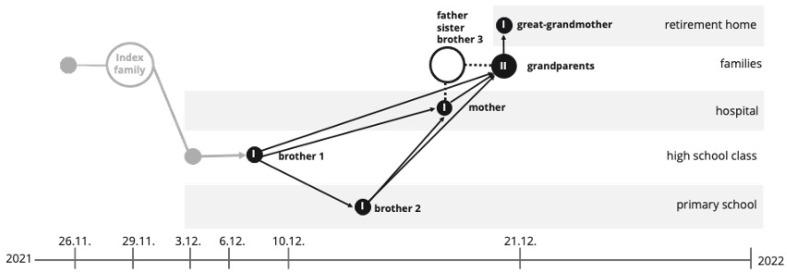
Transmission timeline and pathway of SARS-CoV-2 infections across different biotopes from 26 November 2021 to early January 2022. The diagram illustrates the spread of COVID-19 from an index family through various environments, including a retirement home, hospital, high school, and primary school. Circles represent infected individuals in each setting, with arrows indicating transmission pathways among family members and within institutional environments. Key nodes include the initial infection of a healthcare worker and subsequent spread to patients in the hospital, as well as secondary transmissions within a high school class and primary school.

In the school setting, mask wearing was only mandated indoors, and students spent most of their break times outdoors, where masks were not mandated. By 13 December 2021, all identified SARS-CoV-2-positive students, and their contacts had been isolated, with additional preventive measures promptly enforced. The Christmas holiday period contributed to outbreak containment by reducing school-based transmission opportunities. Ultimately, the outbreak was contained, and all restrictions were lifted 35 days after the outbreak’s initial onset (Figure 4).

#### 3.4.4. Integration of Clinical and Wastewater Monitoring

Wastewater viral genomic surveillance during the study led to the detection of the B.1.640 variant in the inlet of a municipal wastewater treatment plant in the Ruhr metropolitan area. This plant receives wastewater from the northern part of Essen and other surrounding cities, providing a broad population-level snapshot of viral circulation. To contextualize these findings, Table 1 compares wastewater observations with reported clinical cases of the B.1.640 variant at the time of the study both at the national level, as well as at the level of the maximum care hospital in Essen. National clinical data were obtained from the German Robert Koch Institute, as part of their genomic surveillance from symptomatic patients.

We can observe that the B.1.640 variant was identified in wastewater one week before the first clinical case reported in the City of Essen. This suggests that the variant was already circulating within the community before its first clinically detected case, reinforcing the early warning capacity of WBE for detecting emerging variants [24] and further validating the epidemiological significance of these findings.

In linking wastewater surveillance to clinical case reports, these findings highlight the potential of WBE to complement traditional patient-based surveillance, providing a more comprehensive and timely understanding of variant spread within a population.

## 4. Discussion

This study presents a detailed examination of a COVID-19 outbreak caused by the rare B.1.640.2 variant in a German school. It highlights the complexities of variant containment in high-contact settings like schools and underscores the essential role of comprehensive, adaptive hygiene measures [25]. The outbreak’s progression emphasizes the need for rapid, robust preventive strategies to mitigate SARS-CoV-2 transmission, especially for emerging variants whose behaviors are still largely unknown.

The school’s pre-existing hygiene measures, including twice-weekly rapid antigen testing for staff and daily testing for symptomatic individuals, were aligned with standard educational guidelines and provided an effective framework for early detection [26]. However, the outbreak’s dynamics suggest that more intensive strategies, such as mandatory indoor mask wearing within all grade cohorts, enhanced ventilation and that stricter distancing measures might be necessary to manage highly transmissible variants. The combination of asymptomatic spreaders and minimal initial symptoms created an environment conducive to viral propagation, especially in poorly ventilated areas and during break times when distancing and mask wearing were inconsistent [27,28,29].

Control of this outbreak heavily relied on immediate isolation protocols and extensive testing of close contacts, effectively curtailing further spread. These measures proved critical in limiting in-school transmissions, even after superspreading events in which mask wearing and distancing were relaxed. This aligns with findings from other studies, which indicate that consistent mask use and improved ventilation significantly reduce transmission risk, particularly when coupled with comprehensive contact tracing and testing [26,30,31,32].

This outbreak also demonstrated that even non-variants of concern (VOCs) can create substantial disruptions if hygiene protocols are not meticulously applied. Compared to previous variants like Delta, which rapidly impacted various sectors, the B.1.640.2 variant’s effect within the school setting illustrates that rigorous adherence to preventive protocols is necessary for all variant types [33]. In this case, inconsistent indoor ventilation, mask-wearing policies, and distancing contributed to the spread within the outbreak cohort, supporting the need for strict, consistent hygiene measures.

Furthermore, comparing the clinical testing with data from wastewater surveillance demonstrated the presence of this variant in the population before the first confirmed patient. Several WBE studies performed during the COVID-19 pandemic showed the early detection of viral variants in wastewater, as well as suggested higher anticipation of emerging variants when sampling in more minor, intra-urban scales [23,24,34,35]. The identification of the B.1.640 variant in a wastewater treatment plant with input from more than a million people places the question of a higher-than-identified outbreak, with a large enough number of infected people to be detected with this large-scale sampling. This emphasizes the value of combining clinical surveillance with wastewater surveillance to identify potentially otherwise overlooked outbreaks and to direct localized actions [36].

In addition, this study’s findings on the biphasic infection pattern, with a secondary viral load peak shortly after quarantine release, raise questions about variant-specific viral kinetics. This observation could have (policy) implications for quarantine duration and post-quarantine testing requirements to reduce reintroduction risks in schools and households. Furthermore, genomic analysis confirmed the variant’s origin, underscoring the utility of sequencing in distinguishing imported cases and informing containment strategies that can prevent wider community spread.

Strengthened protocols, including enforced mask use, optimized ventilation systems, and cohort isolation policies to mitigate close-contact exposure, would benefit future strategies for managing outbreaks in schools and similar settings [37]. These measures, particularly when coupled with ongoing genomic surveillance, as recommended by the WHO and public health authorities, are vital in identifying and controlling new variant outbreaks and minimizing the risk of cross-border and community spread [38].

In conclusion, this outbreak illustrates that even rare SARS-CoV-2 variants can initiate significant transmission clusters in schools, underscoring the need for proactive, adaptive outbreak management. The findings highlight the critical role of enhanced hygiene protocols, regular testing, and rapid isolation measures in containing variant outbreaks, especially in educational environments with high contact rates and potential for asymptomatic transmission. Adopting such comprehensive measures as part of standard hygiene school protocols may enhance resilience against future SARS-CoV-2 variants, ultimately protecting both students and the broader community.

## 5. Conclusions

Based on this low number of cases, assumptions on the virological, epidemiological, or clinical features of this B.1.640.2 variant are not warranted. However, our findings again show the unpredictability of new emerging SARS-CoV-2 variants and their introduction from abroad. They exemplify the difficulty of controlling such introductions and subsequent spread. These data also strongly support the implementation of genomic surveillance for SARS-CoV-2 in geographical areas and at a country scale.

Continued monitoring is needed to further elucidate the role of young children in the epidemiological assessment of further COVID-19 outbreaks, especially in environments such as DCCs, where close contact between young children could facilitate the spread of SARS-CoV-2. Such surveillance is necessary to guide public health interventions such as the screening, vaccination, and temporary closure of DCCs. Nonetheless, it is important to consider again the impossibility of predicting the emergence of a new variant introduction from abroad and the difficulty of controlling its subsequent spread within a country. It is essential to ensure genomic surveillance worldwide as an implementation of a genomic track system to prevent the eventual passage of this variant and its eventual spread in other regions. It is too early to speculate in this way, but we must be alert to this variant and other concerns that can be introduced in a country. Of course, vaccination and government advice must be considered by people to protect themselves and others.

COVID-19 prevention in schools involves studying in small groups and minimizing student mixing in activities and transportation. Teachers and parents should lead by wearing facemasks, maintaining hand hygiene, keeping physical distance, etc. School attendance should be avoided at any sign of illness. Learning from home may also reduce the need for class attendance. Outdoor classes should also be considered. COVID-19 prevention encompasses avoiding the ‘three Cs’: closed spaces with poor ventilation, crowded places, and close-contact settings. The European Centre for Disease Prevention and Control’s report on air conditioning and ventilation systems and COVID-19 recommends increasing the air exchange rate and outdoor air use and decreasing air recirculation to reduce spread in indoor spaces [39,40]. Finally, appropriate planning for COVID-19 preparedness and prevention for the next school year is essential.

Based on the small number of cases in this outbreak, we cannot draw definitive conclusions regarding the virological, epidemiological, or clinical characteristics of the B.1.640.2 variant. Nevertheless, this event underscores how even rare SARS-CoV-2 variants can emerge unpredictably from abroad and spread rapidly, especially in settings where close contact is common. It also illustrates the difficulty of controlling introductions from other regions before they become established in a new country.

Our investigation highlights the critical value of robust genomic surveillance at the local and national levels, complemented by tools such as wastewater-based epidemiology. Together, these surveillance methods can provide an early warning of emerging variants, guiding timely public health measures such as screening, vaccination, and targeted closures. In particular, close-contact environments—such as DCCs—require heightened vigilance, given their susceptibility to rapid transmission among young children with minimal or asymptomatic illness.

Despite the inherent challenges, standard preventive measures remain essential in limiting viral spread. In schools, this includes studying in small groups, minimizing student mixing, encouraging mask use, ensuring proper hand hygiene, and improving ventilation through strategies such as increasing air exchange rates and reducing recirculation [38,39]. Prompt isolation and remote learning alternatives can help reduce in-person mixing when symptoms arise. Collectively, these measures align with the ‘three Cs’ principle (avoiding closed, crowded, and close-contact settings), which continues to serve as a practical guide for mitigating SARS-CoV-2 transmission in indoor environments.

Moving forward, appropriate planning for COVID-19 preparedness in schools and other high-contact institutions is crucial. Our experience with the B.1.640.2 variant emphasizes that rigorous genomic surveillance, sustained vigilance regarding public health advice, and ongoing vaccination efforts are integral to preparing for future outbreaks—whatever variant may arise next.

## Figures and Tables

**Table 1:** A comparison of clinical cases associated with this variant detected in wastewater samples from University Hospital Essen and national data from Germany (WWTP: wastewater treatment plant). In 2021, no cases of this variant had been reported in Germany.

	Wastewater	Patients	
Week (2021)	WWTP	Hospital	Number of B.1.640 Cases in Germany
43	-	B.1.617.2	no cases
44	B.1.617.2	B.1.617.2	no cases
45	B.1.617.2	B.1.617.2	no cases
46	-	B.1.617.2	no cases
47	B.1.617.2B.1.640	B.1.617.2	1 case
48	B.1.617.2B.1.640	B.1.617.2 B.1.640	5 cases
49	B.1.617.2BA.1	B.1.617.2 B.1.640.	14 cases
50	not analyzed	B.1.617.2 B.1.640.BA.1	7 cases

## Data Availability

The data presented in this study are available on request from the corresponding author. To ensure compliance with data protection policies, access may be granted to qualified researchers for non-commercial scientific purposes upon reasonable request.

## Supplementary Materials

The following supporting information can be downloaded at https://www.mdpi.com/article/10.3390/v17040477/s1: Figure S1: Results of probe-based melting curve analyses to detect SARS-CoV-2 spike gene mutations S371L/S373P (A), L452R (B), and N501Y (C). Viral RNA extracted from five selected schoolchildren and their relatives (GISAID database accession numbers given) was run in parallel with materials obtained from epidemiologically unlinked patients.
