# Correction: Sachse et al. From Entry to Outbreak in a High School Setting: Clinical and Wastewater Surveillance of a Rare SARS-CoV-2 Variant. *Viruses* 2025, *17*, 477

**DOI:** 10.3390/v17121580

**Published:** 2025-12-04

**Authors:** Sven Sachse, Ivana Kraiselburd, Olympia Evdoxia Anastasiou, Carina Elsner, Sarah Christina Goretzki, Stefan Goer, Michael Koldehoff, Alexander Thomas, Jens Schoth, Sebastian Voigt, Rudolf Stefan Ross, Ulf Dittmer, Folker Meyer, Ricarda Maria Schmithausen

**Affiliations:** 1 Institute for Artificial Intelligence (IKIM), University Hospital Essen, University of Duisburg-Essen, 45131 Essen, Germany; sven.sachse@uni-due.de (S.S.); ivana.kraiselburd@uk-essen.de (I.K.); alexander.thomas@uk-essen.de (A.T.); folker.meyer@uk-essen.de (F.M.); 2 Department of Hygiene and Environmental Medicine, University Hospital Essen, University of Duisburg-Essen, 45147 Essen, Germany; stefan.goer@ctde.eurofinseu.com (S.G.); koldehoff@zotzklimas.de (M.K.); 3 Center for Water and Environmental Research, University of Duisburg-Essen, 45141 Essen, Germany; 4 Institute of Virology, University Hospital Essen, University of Duisburg-Essen, 45147 Essen, Germany; olympia.anastasiou@uni-due.de (O.E.A.); carina.elsner@uk-essen.de (C.E.); sebastian.voigt@uk-essen.de (S.V.); stefan.ross@uk-essen.de (R.S.R.); ulf.dittmer@uk-essen.de (U.D.); 5 Department of Pediatrics I, Neonatology, Pediatric Intensive Care, Pediatric Infectiology, Pediatric Neurology, University Hospital Essen, University Duisburg-Essen, 45147 Essen, Germany; sarah.goretzki@uk-essen.de; 6 Emschergenossenschaft Lippeverband (EGLV), 45128 Essen, Germany; schoth.jens@eglv.de

## Missing Citation

In the original publication [1], eleven references were not cited. Among these, references 15–22 are newly added, while references 9, 14, and 23 (which corresponds to the original reference 25) are pre-existing entries that were not previously cited. Due to an oversight by the authors, some numerals appearing in the Materials and Methods as well as the Results sections should have been referenced, but were not marked in square brackets or included in the reference list.

The following references should be included:

9. Colson, P.; Delerce, J.; Burel, E.; Beye, M.; Fournier, P.E.; Levasseur, A.; Lagier, J.C.; Raoult, D. Occurrence of a substitution or deletion of SARS-CoV-2 spike amino acid 677 in various lineages in Marseille, France. *Virus Genes* **2022**, *58*, 53–58. https://doi.org/10.1007/s11262-021-01877-2.
14. Quick, J.; Grubaugh, N.D.; Pullan, S.T.; Claro, I.M.; Smith, A.D.; Gangavarapu, K.; Oliveira, G.; Robles-Sikisaka, R.; Rogers, T.F.; Beutler, N.A.; et al. Multiplex PCR method for MinION and Illumina sequencing of Zika and other virus genomes directly from clinical samples. *Nat. Protoc.* **2017**, *12*, 1261–1276. https://doi.org/10.1038/nprot.2017.066.
15. MEGA (Molecular Evolutionary Genetics Analysis). Available online: https://www.megasoftware.net/ (accessed on 13 December 2023).
16. Thomas, A.; Battenfeld, T.; Kraiselburd, I.; Anastasiou, O.; Dittmer, U.; Dörr, A.K.; Dörr, A.; Elsner, C.; Gosch, J.; Le-Trilling, V.T.K.; et al. UnCoVar: A reproducible and scalable workflow for transparent and robust virus variant calling and lineage assignment using SARS-CoV-2 as an example. *BMC Genom.* **2024**, *25*, 647. https://doi.org/10.1186/s12864-024-10539-0.
17. O’Toole, Á.; Scher, E.; Underwood, A.; Jackson, B.; Hill, V.; Mccrone, J.T.; Colquhoun, R.; Ruis, C.; Abu-Dahab, K.; Taylor, B.; et al. Assignment of Epidemiological Lineages in an Emerging Pandemic Using the Pangolin Tool. *Virus Evol.* **2021**, *7*, veab064.
18. Bray, N.L.; Pimentel, H.; Melsted, P.; Pachter, L. Near-optimal probabilistic RNA-seq quantification. *Nat. Biotechnol.* **2016**, *34*, 525–527.
19. Khare, S.; Gurry, C.; Freitas, L.; Schultz, M.B.; Bach, G.; Diallo, A.; Akite, N.; Ho, J.; Lee, R.T.; Yeo, W.; et al. GISAID’s Role in Pandemic Response. *China CDC Wkly.* **2021**, *3*, 1049–1051. https://doi.org/10.46234/ccdcw2021.255.
20. Garrison, E.; Marth, G. Haplotype-based variant detection from short-read sequencing. *arXiv* **2012**, arXiv:1207.3907.
21. Rausch, T.; Zichner, T.; Schlattl, A.; Stutz, A.M.; Benes, V.; Korbel, J.O. DELLY: Structural variant discovery by integrated paired-end and split-read analysis. *Bioinformatics* **2012**, *28*, i333–i339.
22. Köster, J.; Dijkstra, L.J.; Marschall, T.; Schönhuth, A. Varlociraptor: Enhancing sensitivity and controlling false discovery rate in somatic indel discovery. *Genome Biol.* **2020**, *21*, 98. https://doi.org/10.1186/s13059-020-01993-6.
23. Wilhelm, A.; Widera, M.; Grikscheit, K.; Toptan, T.; Schenk, B.; Pallas, C.; Metzler, M.; Kohmer, N.; Hoehl, S.; Marschalek, R.; et al. Limited neutralisation of the SARS-CoV-2 Omicron subvariants BA.1 and BA.2 by convalescent and vaccine serum and monoclonal antibodies. *EBioMedicine* **2022**, *82*, 104158. https://doi.org/10.1016/j.ebiom.2022.104158.

These eleven references have now been inserted in the following sections: Section 2.3.2. Molecular Characterization of SARS-CoV-2 Isolates, Section 2.3.3. Next-Generation Sequencing and Data Analysis, Section 2.3.4. Viral Detection in Wastewater, and Section 3.1. Laboratory Results. The main text should now read as follows:

Section 2.3.2:

“The sequence editing, generation, and translation of the consensus sequence into the corresponding amino acid sequence were performed using Geneious Pro 5.1.7. A multiple-sequence alignment was performed by Clustal Omega version 2.1 using the SARS-CoV-2 isolate Wuhan-HU-1 (NC_045512.2) as a reference. The visualization of the Clustal Omega alignment was carried out with a Highlighter analysis using the Los Alamos National Laboratory pathogen database. Initial manual lineage prediction was performed based on mutations identified in a multiple-sequence alignment using out-break.info [14]. Whole-genome sequencing (see below) later confirmed the initial lineage assignment.”

“A phylogenetic tree was inferred using the Maximum Likelihood method and the Tamura-Nei nucleotide substitution model based on the receptor binding domain of the viral spike gene (nucleotides 963 to 1737). The tree was drawn to scale, with branch lengths measured in the number of substitutions per site. Statistical robustness was tested using the bootstrap approach with 1000 replicates. Analysis was conducted in MEGA, version 11 [15].”

Section 2.3.3:

“Reads with an average Phred quality below 20 and a length below 30 base pairs were excluded, enabling downstream analysis. Subsequently, data analysis was performed with the UnCoVar bioinformatic pipeline for reconstructing whole viral genomes [16]. UnCoVar performed a series of QC steps, initially attempted de novo assembly, and then resorted to co-assembly for recalcitrant samples; it subsequently used pangolin [17] and Kallisto [18] matching to GISAID [19] to obtain lineage calls. Additionally, Freebayes [20], Delly [21], and Varlociraptor [22] were utilized for variant calling.”

Section 2.3.4:

“During this study, wastewater samples were collected within the metropolitan area Ruhr and processed for SARS-CoV-2 detection, as described [23]. The sampling area comprises both the high school and maximum care hospital described in this work.”

“Viral variants were identified with a modified version of UnCoVar [16], based on the detection of variant-specific mutations without the attempt at genome assembly.”

Section 3.1:

“This characteristic pattern of amino acid exchanges had already been reported for variants from the former SARS-CoV-2 B.1.640 lineage, which was renamed to B.1.640.1 [9]. Analyses of full-lengths sequences obtained by next-generation sequencing confirmed and extended the findings from [9]. Additionally, the formation of a so-called monophyletic group in a tree based on partial SARS-CoV-2 spike gene RBD sequences, which reconstructs the evolutionary history of the viral isolates (Figure 2), shows that the children and their relatives were infected in a single-source outbreak by a yet rare SARS-CoV-2 B.1.640.1 variant, which had possibly originated in the Republic of Congo [9].”

With the above corrections, the reference citation numbers 15–32 have been changed to 15–40, respectively. The authors state that the scientific conclusions are unaffected. This correction was approved by the Academic Editor. The original publication has also been updated.
