# Supplementary material for: From Entry to Outbreak in a High School Setting: Clinical and Wastewater Surveillance of a Rare SARS-CoV-2 Variant
Source: Viruses. 2025 Mar 27;17(4):477. doi: 10.3390/v17040477 (PMC12030855; doi:10.3390/v17040477)
Supplement: Supplementary file 1 [file viruses-17-00477-s001.zip › viruses-3501151-supplementary.pdf]

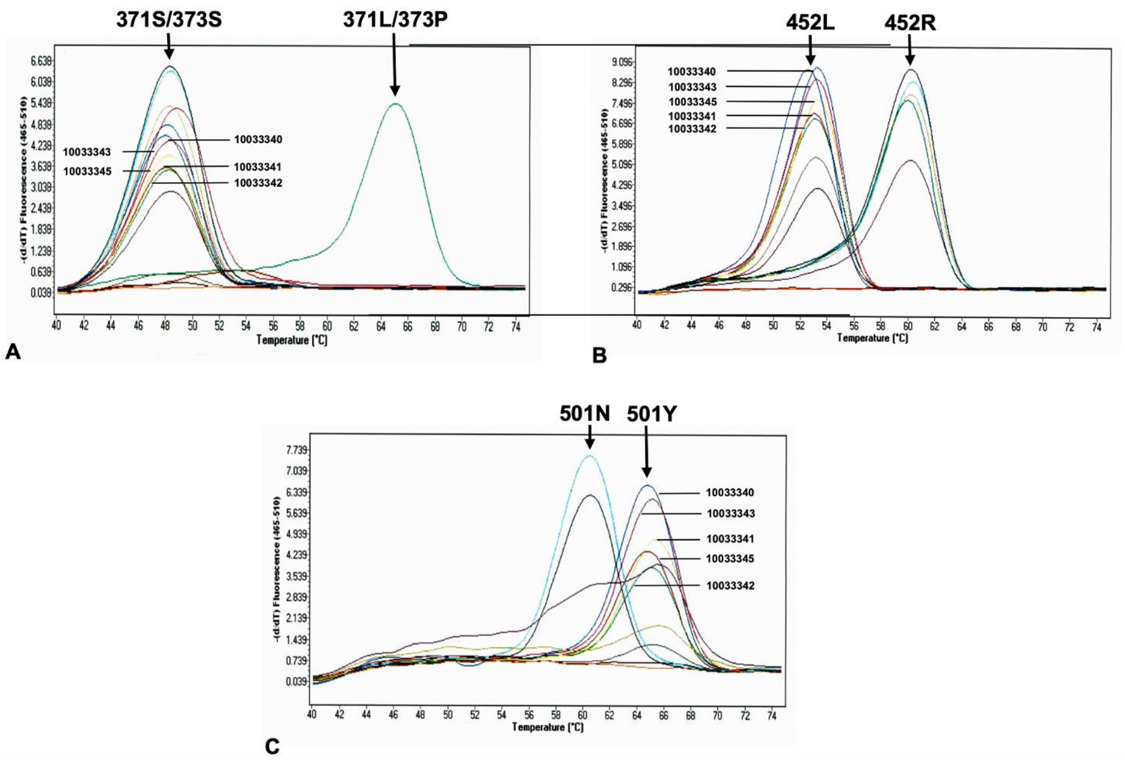

Figure S1. Results of probe-based melting curve analyses to detect SARS-CoV-2 spike gene mutations S371L/S373P (A), L452R (B), and N501Y (C). Viral RNA extracted from five selected schoolchildren and their relatives (GISAID database accession numbers given) was run in parallel with materials obtained from epidemiologically unlinked patients.
